# A critical review on advances in the practices and perspectives for the treatment of dye industry wastewater

**DOI:** 10.1080/21655979.2020.1863034

**Published:** 2020-12-28

**Authors:** Toral Shindhal, Parita Rakholiya, Sunita Varjani, Ashok Pandey, Huu Hao Ngo, Wenshan Guo, How Yong Ng, Mohammad J. Taherzadeh

**Affiliations:** a Paryavaran Bhavan, Gujarat Pollution Control Board, Gandhinagar, India; b Biotechnology Department, Kadi Sarva Vishwavidyalaya, Gandhinagar, India; c Centre of Innovation and Translation Research, CSIR-Indian Institute of Toxicology Research, Lucknow, India; d Centre for Technology in Water and Wastewater, School of Civil and Environmental Engineering, University of Technology Sydney, Sydney, Australia; e Department of Civil & Environmental Engineering, National University of Singapore, Environmental Research Institute, Singapore, Singapore; f Swedish Centre for Resource Recovery, University of Borås, Borås, Sweden

**Keywords:** Industrial process, dye industry wastewater, waste generation, advanced wastewater treatments, microbial degradation, resource recovery

## Abstract

Rapid industrialization has provided comforts to mankind but has also impacted the environment harmfully. There has been severe increase in the pollution due to several industries, in particular due to dye industry, which generate huge quantities of wastewater containing hazardous chemicals. Although tremendous developments have taken place for the treatment and management of such wastewater through chemical or biological processes, there is an emerging shift in the approach, with focus shifting on resource recovery from such wastewater and also their management in sustainable manner. This review article aims to present and discuss the most advanced and state-of-art technical and scientific developments about the treatment of dye industry wastewater, which include advanced oxidation process, membrane filtration technique, microbial technologies, bio-electrochemical degradation, photocatalytic degradation, etc. Among these technologies, microbial degradation seems highly promising for resource recovery and sustainability and has been discussed in detail as a promising approach. This paper also covers the challenges and future perspectives in this field.

## GRAPHICAL ABSTARCT

**Figure:**
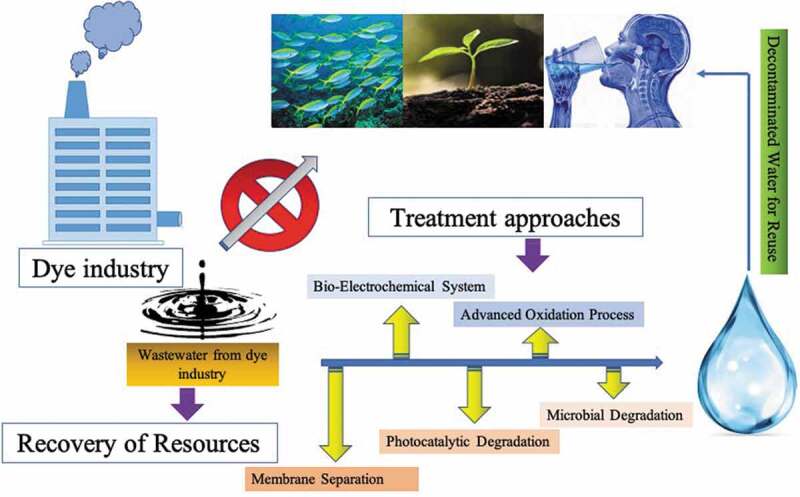

## 1. Introduction

Rapid progress in industrialization in last few years has increased discharge of pollutants into environment [1,2]. Discard of municipal and other industrial wastes into water bodies causes water pollution [3,4]. Industries release non-treated and/or incompletely treated wastewater in the environment which leads water and soil pollution. Mostly pharmaceutical, textile, food, paper & pulp and cosmetic industries uses synthetic dyes for manufacturing process. Dyeing procedure of textile industry uses a massive volume of water in fixing, dyeing, and washing procedures [5]. The dyes are soluble organic compounds, specifically those classified as direct, reactive, acidic and basic [6]. Dyes exhibit high solubility in water and make it more difficult to be removed by conventional procedures [7].

The atmosphere is mainly polluted by release of crude and unprocessed waste in the ecosystem which mainly contains majority emissions produced by industrial activities [8–10, 117]. Textile industries emit greenhouse gases by manufacturing processes and release hazardous gases and pollutants which are harmful for human, and plants [11]. Most dyes contain mutagenic, toxic, and carcinogenic properties [12,13].

Industrial dyes as well as their by-products are main contaminants that are carcinogenic and toxic. Hence, they lead harmful effects on ecosystem [14]. Various reports are available which shows harmful effects of azo dyes on plants (plant growth and germination) [15–18].

Industrial pollutants are one of the biggest environmental problems due to their effect on groundwater and surface water along with human health [2,19,20]. Various technologies such as advanced oxidation process, membrane filtration technique, microbial technologies, bio-electrochemical degradation, photocatalytic degradation have been reported for treatment of dye industry wastewater [3,21–24]. This paper presents and discusses advanced technical and scientific developments about dye industry wastewater treatments. This paper focus on microbial degradation of dyes as it has been reported as highly promising for resource recovery and sustainability. This paper also includes the bottlenecks and perspectives in this field of research.

## 2. Dye industry processing

The dye industrial wastewater treatment procedures are a unique key feature in effective trading of dye industry products. The dye industries produce fibers that are further converted into yarn and lastly transformed into textile materials. These materials are prepared by using steps of wet processing. In the textile coloring procedure, unsettled dye gets wash out with the water. Dry procedure produce large amount of solid waste as compared to the wet procedure.

Industrial manufacturing procedure includes basic steps for industrial processing as narrated below. Mainly, industrial procedure releases different types of pollutants like dyes, metals, crude oil and hydrocarbons, etc.

### 2.1. Sizing and desizing

Sizing is an important process that helps to increase strength of yarn and decrease breakage of yarn as well as scuffs its resistance. Sizing acts as a glaze or defensive material to adjust physical appearance of constituents. Selection of sizing agents is based on environmental friendliness, types of fabric, effluent treatments, cost-effective, easy removal, etc. Most sizing agents are natural elements like starch, protein-based starch, by-products of cellulose, etc., and other synthetic agents like polyacrylates, PVA (Polyvinyl Alcohol), Acrylic resins and polyesters, etc [25,26]. Starch is used in sizing procedure and it is degradable in H_2_O and CO_2_ by oxidation process and desizing enzymes can be converted into ethanol. Ethanol is additionally used as an energy source and also reduce BOD level in treatment process [27].

Desizing is a procedure of eliminating the sizing agent from textile fabrics and blends to prepare the fabric for further processing. Starch and its derivatives are mostly used because of their easy availability, brilliant film forming capacity, and relatively low cost. Completion of this procedure can be carried out by using enzyme desizing, acid desizing, oxidative desizing, or by removing water-soluble sizes. α-amylase is used to solubilize starch by hydrolyzing starch at pH between 5.5 to 7.5 in enzymatic desizing of textile fabrics [28]. Desizing process lower pH of the waste around 4–5 pH with a higher BOD (Biological Oxygen Demand) (300–450 ppm). Desizing processing step release glucose, resins, waxes, PVA, volatile organic compounds, starch and fats, etc [29].

### 2.2. Scouring

Chemical washing procedure is known as scouring. This method is used to eliminate contaminations like surfactants, waxes, fatty acids and natural oils from fibers by bridge scour, hydrodynamic scour, ice scour and tidal scour [26,30]. Detergents, NaOH, oils, fats, spent solvents, wax, surfactants, alkylbenzene sulfonates, alkylphenol and ethoxylates are compounds released during scouring process [31].

### 2.3. Bleaching

In this procedure, undesirable color or dye can be removed from fibers by using different chemicals like sodium hypochlorite, peracetic acid, chlorine, hypochlorite, and hydrogen peroxide. The natural color substance gives a creamy look to the fabrics. Peracetic acid has been reported as more beneficial because it is less destructive to yarn and elevated sheen. Bleaching processing step release hypochlorite, caustic soda, acids, chlorine, hydrogen peroxide, etc [32].

### 2.4. Mercerization

Mercerization is a treatment process that includes use of strong alkaline (18–24% by weight) solution for yarn materials to improve shine of the material. This process involves several steps such as (i) dipping yarn fabrics into highly concentrated alkaline solution, (ii) to remove alkali, fabric is placed into the water. This process improves fabric absorbing capacity, smoothness, strength and reaction capability with various chemicals. Mercerizing step releases caustic soda, ZnCl_2_, cyclohexanols and it also increses pH of wastewater [33,34].

### 2.5. Dyeing and printing

The dyeing procedure involves addition of color to material and treatment with dye. The most required factors in this procedure are temperature and time. The effluent from printing and dyeing both involve presence of waste materials. Dyeing step releases reducing agents (sulfides), dyestuff, soap, mordant, acetic acids, metal salt, cationic materials, surfactants and chromophores in wastewater [35,36].

### 2.6. Finishing

Finishing process in textile manufacturing industry transforms knitted or woven material into usable material with better-quality and certain properties like waterproofing and smoothness. This final procedure mostly participates in water pollution [36,37]. Finishing processing step release salts, traces of starch, special finishes, tallow, etc [34].

## 3. Characteristics of dye industry wastewater

The effluent discharged from dye industries contain a mixture of metals, dyes and other pollutants. Two types of industrial wastes (liquid and solid) are frequently formed due to manufacturing processes and they contain any material that can be reduced through product manufacturing procedure. Liquid waste (i.e. wastewaters released from industries) is dangerous to existing living organisms as well as to the environment as it also carries different types of toxic contaminants. However, the characteristics and nature of industrial effluent are based on type of manufacturing process and products [38].

The wastewater produced through the wet process from dye industries is marked by a high level of dissimilarities in many parameters like chemical oxygen demand (COD), pH, total solids (TS), biological oxygen demand, water usage, and color. Wastewater having 0.25 BOD/COD ratio determines that the industrial wastewater having vast actions of organic material which is non-biodegradable. Composition of industrial wastewater is based on various types of raw materials, chemicals, organic-based compounds and different types of dyes used in wet and dry processing phases. The industrial manufacturing procedure discards unsafe and colored dyes mostly azo dyes. These dyes are a vital base of environmental pollution that causes harmful effects on aquatic life because of its low biodegradability, strong color, high COD and BOD. Despite the pollutants, dye industrial wastewater contains adjustable ionic strength, high salt concentration, and pH variations.

Process waste is generated during different industrial manufacturing procedures. This type of waste is highly polluted and contains high COD and BOD levels, color pigments, pH, high salt concentration, etc [39,40].

The dyes can be classified as synthetic dyes and natural dyes. The synthetic dyes are easily produced in different colors and characterized by their fastness, so it is more widely used dye as compared to natural dyes. Synthetic dyes are classified into different groups (i) based on their mode of application like direct, reactive, basic, disperse and vat dying, etc. and (ii) based on their chemical structure like anthraquinone, azo, phthalocyanine, sulfur and triarylmethane, etc [41].

The azo dyes are characterized by comparatively high recalcitrance and high polarization. When particles contain other acidic groups like carboxyl, hydroxyl or sulfonyl substituents azo dyes can be categorized by amphoteric properties. The azo dyes are cationic, anionic, or nonionic. Existence of an amino group causes high water solubility and high mobility in comparison with hydrocarbons, high boiling point and a lower Henry’s law constant [42].

### 3.1. Effects of azo dyes on plants and human health

Textile industries release number of hazardous pollutants and which are toxic and carcinogenic effects in their nature [43]. Hence, they cause environmental degradation and various diseases in humans, animals and plants [44]. Red-S3B (3.19% N) azo dye is the most toxic to growth and germination of 7 days old wheat saplings. *Aspergillus terreus* NIAB-FM10 and *Shewanella sp*. NIAB-BM15 were found to be more effective for degradation of Red-S3B. The mixture of these two species give complete decolorization of Red-S3B (500 mg) in 4 hour. After treatment with industrially important consortium increased shoot length and root length, shoot biomass, root biomass of 30 day old wheat saplings were noted [45].

Sudan I dye (Solvent Yellow 14) belongs to the family of azo-lipophilic complexes commonly used in industrial segments [46]. When they present in the humans and animal bodies, it is enzymatically transformed into carcinogenic aromatic amines through the action of the intestinal flora [47].

Basic Red 9 dye having high environmental toxicity and carcinogenicity, it breaks down under anaerobic condition into carcinogenic aromatic amines and have potential for skin irritation, cancer itself, allergic dermatitis, and mutations [48–51].

## 4. Treatment process for dye industry wastewater

**Figure 1.**
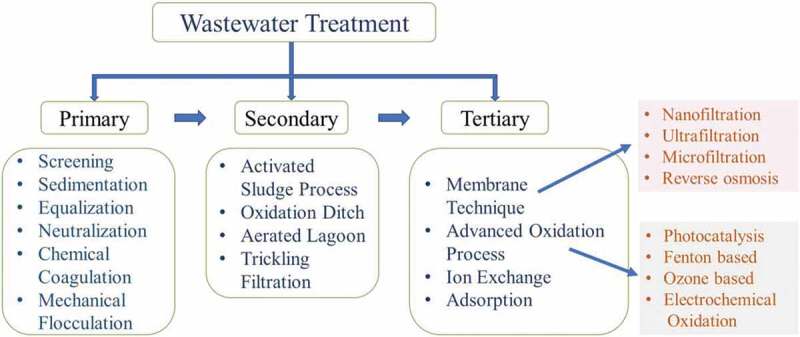
Classification of wastewater treatment

The dye industrial wastewater treatment process is mainly classified into three types: (i) biological, (ii) chemical and (iii) physical treatments. General wastewater treatment includes preliminary treatment, primary treatment, secondary treatment and tertiary treatment to treat dye industry effluents. Preliminary treatment includes neutralization and equalization [34]. The primary treatment includes sedimentation, screening, chemical coagulation, flocculation and floatation. Secondary treatment includes chemical/physical separation or biological oxidation and used to reduce organic compounds. Tertiary treatment is more significant than others because it enhances effluent treatment [52]. Figure 1 shows classification of wastewater treatment types.

### 4.1. Preliminary treatment

The first step in wastewater treatment is equalization and mixing wastewater streams that were discharged at different intervals from different stages during the manufacturing process. Equalization confirms that the waste has a uniform characteristics in terms of pH, pollution load and temperature [52].

### 4.2. Primary treatment

The primary treatment involves screening, sedimentation and floatation. However, by use of steps above some suspended, fine and colloidal elements cannot be efficiently removed [53]. In some cases, chemical coagulation and mechanical flocculation is employed [52]. In chemical coagulation, addition of coagulants like ferrous sulfate (FeSO_4_), polyelectrolyte, alum, ferric chloride (FeCl_3_), lime [Ca(OH)_2_] are used for flash mixing. This procedure was carried out on flocculation and settling tank or in clariflocculator. Iron salts and aluminum are commonly used as coagulants in water and wastewater treatment [54]. Chemical treatments are used to reduce suspended solids, color, COD and BOD. Chemical coagulation procedure efficiently decolorizes insoluble dyes, but it is not very much effective in reduction of soluble dyes [55,56].

### 4.3. Secondary treatment

In secondary wastewater treatment, color and dissolved or colloidal organic material present in dye wastewater is stabilized [57]. This procedure is carried out with the help of fungi, bacteria, algae, yeast and other microbes. This procedure can be anaerobic or aerobic. In aerobic procedure bacteria, algae and some other microbes utilize organic compounds as food and show successive modifications: (i) Flocculation and coagulation of colloidal compounds, (ii) Oxidation of dissolved organic compounds to CO_2_, and (iii) Degradation of nitrogenous organic compounds to ammonia, which is then converted into nitrite and then nitrate. Anaerobic treatment is mostly used for digestion of waste. The capability of this procedure depends on temperature, pH, absence of oxygen, waste loading and presence of toxic material. Aerobic treatment for azo dyes has proven unsuccessful in maximum cases however, nowadays it is used as typical treatment methods [52,55,56].

### 4.4. Tertiary treatment

The dye industrial wastewater contains various types of hazardous dyes. They require advanced treatment method or tertiary treatment to remove particular pollutants. Generally, tertiary treatment is used to remove organic color compounds by adsorption and dissolved solids by membrane filtration techniques. The wastewater can be treated with ozone (O_3_) or another oxidizing agent to destroy many contaminants [52].

## 5. Treatment methods

The recent treatment approaches for dye industrial wastewater include membrane separation, adsorption, advanced oxidation procedures (AOPs), bio-electrochemical treatments and photocatalytic degradation for reduction of organic pollutants from industrial effluent [58–60]. Table 1 depicts treatment technologies for dye industrial wastewater.

**Table 1.** Treatment technologies for dye industrial wastewater

Sr. no.	Technology	Applications/Characteristics	References
1.	Enzymatic biodegradation [Laccase)	Decolorization of textile wastewater and textile dyes Detoxification of phenols and chlorophenols	[114]
2.	Photocatalytic degradation [Photocatalytic membrane]	Removes dyes from textile wastewater Removes industrial pollutants from textile wastewater	[115]
3.	Photocatalytic degradation [Intimate coupling of photocatalysis and biodegradation]	Achieves rapid treatment rate and high efficiency of pollutants removal under the action of microorganism and photocatalytic reaction	[116–118*]
4.	Bio electrochemical system	Removes pollutants from dye industrial wastewater with simoultaneous recovery of resources such as power/energy and heavy metals etc.	[119]
5.	Enzymatic degradation [Azoreductase]	Azoreductase enzyme breaks down azo compounds and azo dyes through reductive cleavage of azo bonds	[114]

### 5.1. Membrane techniques

Membrane separation is an advanced technology for wastewater treatment. In this process, wastewater is allowed to pass through a porous membrane. If any solute is bigger than membrane pore size than it will be trapped and rest of the solution will pass through the membrane. The trapped solutes from filter cake or layer, are removed constantly during the filtration procedure. The membrane separation procedures are classified based on size of porous membranes.

Pressure-driven membrane procedures can be divided into four main classes, (i) RO (Reverse osmosis), (ii) UF (Ultrafiltration), (iii) MF (Microfiltration) and (iv) NF (Nanofiltration) [61]. NF membranes contain 0.1 to 10 mm pore size with the lowest applied pressure. UF membranes contain 2 to 100 nm pore size with high applied pressure and low water permeability. MF and UF involve the same sieving mechanism, which is innovative and sustainable technology [62]. UF is used for the recycling and separation of water-insoluble dyes such as disperse dye and indigo dye, whereas NF and RO procedures are used to hydrolyze reactive dyes from dye wastewater. MF is generally not used for wastewater treatment because of their large pore size [63,64]. Type of membrane filter used for separation depends on numerous factors like nature of dye, dyeing process and chemical composition of pollutants, etc. Membrane used for reverse osmosis and ultra-filtration are generally prepared from different polymers like polyacrylonitrile, polysulphonates, polycarbonate, polyamides, fluorocarbon-based polymers and polypropylene etc [32,56].

#### 5.1.1. Ultrafiltration

Ultrafiltration technique requires lower pressure than reverse osmosis and nanofiltration, thus make it more efficient. Polyether sulfone (PES) membrane is used for removal of dyes from wastewater. PES contains 1kDa and 10kDa porous membranes used for dyes removal. 1kDa polyether sulfone (PES) membrane gives 80% to 100% dye removal whereas 10kDa polyether sulfone (PES) membrane is not useful for the removal of dyes. The ultrafiltration technique is more suitable to be used as a pre-treatment procedure [65].

#### 5.1.2. Nanofiltration

NF shows higher permeability and low transmembrane pressure. The energy consumption in NF is lower than MF [66]. Hence, NF is one type of competitive technique to treat dye industrial wastewater. In the past decade many researchers have focused on removal of reactive dyes from dye industrial wastewater. Eg. [67], found the rejection of Reactive Black-5 up to 98% by Nanofiltration membranes. [68], reported 99.5% removal of reactive brilliant blue (KN-R) from dye industrial wastewater using ES404 polyether-sulfone (PCI, UK) membrane.

#### 5.1.3. Microfiltration

This method is used as a pre-treatment procedure for nanofiltration or reverses osmosis. Microfiltration with 0.1–1 μm porous membrane was used for removal of dye pigments from the dye industrial effluent [35,61].

#### 5.1.4. Reverse osmosis

Reverse osmosis procedure is used to eliminate chemical compounds as well as decolorization of different dyes from dye wastewater. Decolorization and removal of chemical complexes from dyehouse wastewater can be passed out in a single phase of reverse osmosis procedure. RO membrane has 90% retention rate for most types of ionic compounds. Reverse osmosis supports elimination of chemical compounds, hydrolyzed reactive dyes and minimal salts [35,61].

### 5.2. Advanced oxidation processes

The advanced oxidation process (AOP) has been reported as one of the efficient procedure to reduce organic pollutants from dye industry effluent from the environment and improving availability of organic contaminants free water for humanity. AOP is a most suitable technique for degradation of organic dyes by radiation of visible light due to its eco-friendly nature, complete degradation, low cost, increase reusability of water and decrease in the pollutant load [69–71].

#### 5.2.1. Combined bio-advanced oxidation process

72, reported that combined biological treatment with AOPs treatment (Bio-AOP) gives 100% decolorization of Remazol Red (RR), Reactive Black 5 and Reactive Red 180 (RR 180) by *Aeromonas hydrophila* SK16. However for individual treatment 72% decolorization rate was reported. Combination of AOPs treatment with biological treatment is more effective than single wastewater treatment.

#### 5.2.2. Photocatalytic degradation

Photocatalytic degradation has been reported for treatment of dye containing wastewater. Heterogeneous photocatalytic oxidation is one of the most important technique among the AOPs which is commonly known as photo-catalysis [73,74]. Semiconductor nanoparticles are used for photocatalytic degradation of organic contaminants. Many oxides contain suitable bandgap for photocatalytic reactions. TiO_2_ (Titanium Dioxide) is used in photocatalytic degradation due to its nontoxic nature, chemical stability and environmental compatibility [24]. The use of TiO_2_ has been recognized as a proficient AOP and versatile because of its ability to produce reactive oxidative species (like O_2_- and •OH, ROS) which is highly desired for photocatalytic degradation of organic pollutants and dyes [75]. The combination of coagulation and flocculation procedure with TiO_2_- modified UF membrane gives best results for degradation of reactive black 5 dye [76].

[77], have reported photocatalytic experiments and noted that Zeo-TiO_2_ mixture gives best result in photocatalytic performance on degradation of Rhodamine B dye than Zeo-ZnO composites under UV radiation. Zeo-TiO_2_ catalyst has excellent recycling stability.

Reactions responsible for photocatalytic degradation of dye can be written as below [78].

(1) $$Photocatalyst+h\nu \to hVB^{+}+eCB$$

(2) $$O_{2}+eCB\to \cdot O_{2}\left(Superoxideradical\right)$$

(3) $$H_{2}O+hVB^{+}\to \left(hydroxylradical\right)+H^{+}$$

(4) $$hVB^{+}+eCB\to Energy\left(Heat\right)$$

(5) $$O_{2}+Pollutant\to Intermediates\to \;H_{2}O+CO_{2}$$

(6) $$OH+Pollutant\to Intermediates\to \;H_{2}O+CO_{2}$$

In photocatalytic degradation technique, UV radiation is required for photocatalytic activation. Thus, TiO_2_ photocatalysts show low absorption in visible light and because of that efficiency of TiO_2_ interrupts in natural sunlight. Light absorbing properties of TiO_2_ can be spread by doping TiO_2_ with metals. Correct doping can improve photo-reactivity of TiO_2_ in both visible and UV light [79–81].

Factors that are responsible for different photocatalytic action of manufactured materials are the nature of cations (Ni, Cu), irradiation source, light power and photo-catalyst synthesis methods [82]. Nickle and copper metal ions are generally used as bimetallic substance and have been described as the effective process to improve effectivity of many reactions, such as (i) Cu and Ni for photocatalytic water splitting [83], (ii) Cu TiO_2_/ZnO for Methyl Orange (MO) [14,23,84], (iii) doping TiO_2_ with low concentrations of Co and Ni or Fe and Cu recovers its photocatalytic effectiveness [85]. Literature is available which shows use of Cu or Ni-based TiO_2_ photocatalysts for degradation of dye, like methylene blue and methyl orange under UV irradiation [14,23,84].

### 5.3. Bio-electrochemical system

**Figure 2.**
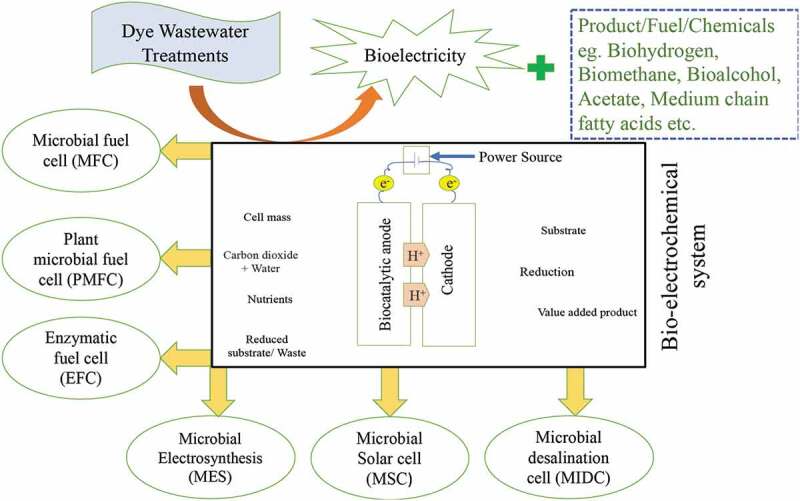
Schematic presentation of different types of bio-electrochemical systems (BES) and their application

BES is a developing technology to improve energy and environment relevant problems by making wastewater treatment procedures more sustainable and more economical. The bio-electrocatalytic reaction combined with extracellular electron transfer can drive several procedures such as synthesizing chemicals, producing electricity from wastewater, removing pollutants and desalinating seawater [86,87]. Removal of untreated wastewater from different industries such as textile, dyestuff, paper, etc., involves 70% identified commercial dyes which includes common chromophores in reactive dyes. Bio-electrochemical systems (BESs) contain great potential for azo dye removal [88]. [88], used bio-electrochemical systems to decrease the concentration of Reactive Black 5 (RB5) from 0.503 ± 0.002 mM to 0.124 ± 0.007 mM after 10 h of operating. Figure 2 shows schematics of different types of bio-electrochemical systems (BES) and their applications.

### 5.4. Microbial degradation

Various microorganisms like bacteria, algae, fungi and yeast can be used for dye degradation [89].

#### 5.4.1. Bacterial biodegradation

Many bacterial strains are used to degrade dyes in aerobic or anaerobic conditions [90]. *Pseudomonas luteola, Xanthophilus azovorans, Klebsiella pneumonia, Clostridium perfringens*, are used in azo dye degradation. The genetically engineered *E. coli* strain gives increased azo reductase activity [91].

[92], has found that the *Aeromonas hydrophila* LZ-MG14 was able to degrade 96.8% (200 mg L^−1^) malachite green (MG) within 12 h from dye industry wastewater. Bioaugmentation by *Aeromonas hydrophila* LZ-MG14 in a membrane bioreactor [MBR) improved the efficiency of malachite green degradation. [93], have reported that *Pseudomonas aeruginosa* and *Bacillus subtilis* can reduce 92.13% and 88.21% Allura Red (R-40] dye respectively, under microaerophilic conditions. *Halomonas sp*. strain was isolated from coastal sediments which was contaminated by chemical wastewater and was found to give 90% azo dye degradation in 24 hours using yeast extract as a carbon source at temperature 30° C. The result showed that bacterial strain decolorizes different azo dyes in higher saline conditions [94].

[95], studied the complete degradation of Methyl Orange (sulfonated azo dye) by *Bacillus stratospheric* SCA1007. *Bacillus stratospheric* SCA1007 gave comprehensive degradation of Methyl Orange (150 mg/L) in a different range of dye concentration, at 7 pH and 35°C temperature under static condition. The degradation of azo dyes was studied by Fourier Transform Infrared Spectroscopy (FTIR) and Ultra-violet Visible spectroscopy (UV-vis). Toxicity studies were done on *Vigna radiata* and *E. coli* to check the nontoxic nature of degraded products. Table 2 shows degradation of different dyes using bacteria.

**Table 2.** Degradation of different dyes using bacteria

Sr. No.	Name of dye	Dye degrading bacteria	References
**1**	Reactive Red 198	*Klebsiella variicola – Enterococcus faecalis*	[120]
**2**	Reactive Violet 5	*Staphylococcus aureus*	[121]
**3**	Reactive Red 239	*Bacillus* sp. strain CH12	[122]
**4**	Congo red	*Brevibacillus parabrevis*	[123]
**5**	Indigo carmine	*Geobacillus stearothermophilus*	[124]
**6**	Azure B dye	*Bacillus sp*. MZS10	[125]
**7**	Hair dye	*Enterobacter cloacae* DDB I	[126]
**8**	Orange 3 K	*Bacillus* sp.	[127]
**9**	Orange M2R	*Bacillus farraginis*	[128]
**10**	Reactive Red 239	*Bacillus* sp. strain CH12	[122]
**11**	Metanil yellow [sulfonated azo dye]	*Lysinibacillus* sp. AK2	[129]
**12**	methyl orange	*M. yunnaenensis*	[130]
**13**	Direct Red 81 [DR81]	*Bacillus sp*. DMS2	[131]

#### 5.4.2. Fungal biodegradation

Filamentous fungi can grow on range of ecological niches like living plants, soil and organic waste because of their speedy adaptation and metabolism on varying nitrogen and carbon sources. Fungi produce a huge quantity of extracellular and intracellular enzymes with degrading capability of many types of organic contaminants, like dye effluents, organic waste, steroid compounds and polyaromatic hydrocarbons. Various studies have reported on biodegradation of azo dyes by using white-rot fungi [32]. Mycoremediation has been reported as a safe, low-cost and natural procedure for dye removal [96].

**Enzymes used for textile dye degradation**: The use of enzymes for degradation of textile dye is the most popular method. Triphenylmethane dyes come from the most significant group of synthetic dyes and are used extensively in textile dye industries. They are usually included as xenobiotic compounds. *Penicillium ochrochloron* decolorizes cotton blue dye within 2 hrs under static condition at temperature 25 °C and 6.5 pH.

**Figure 3.**
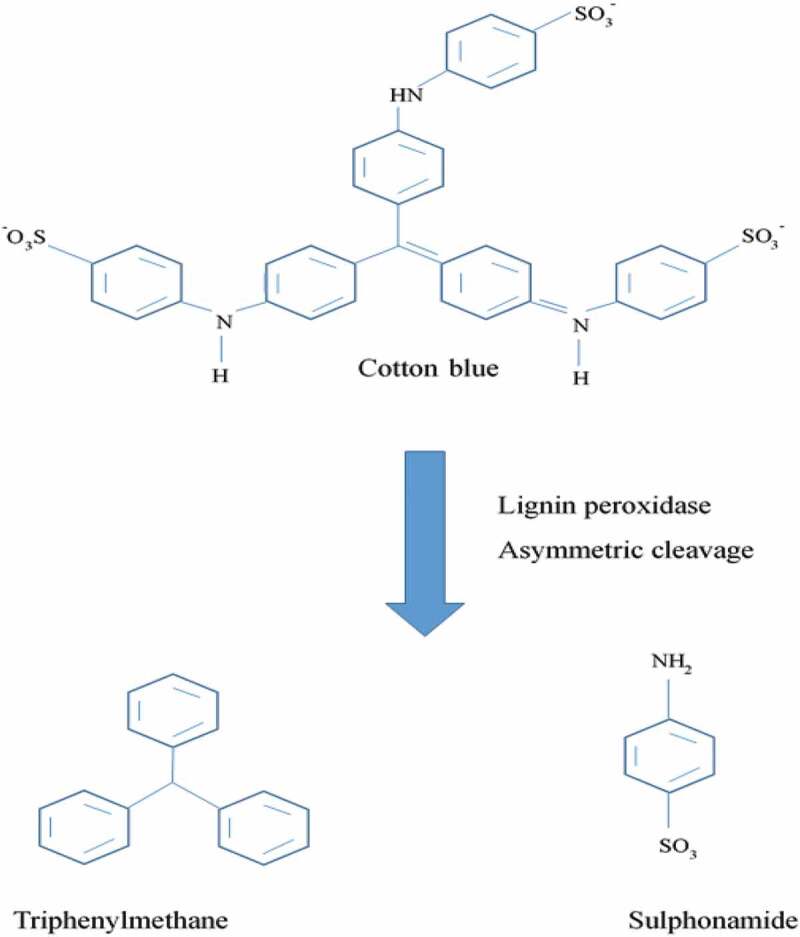
Pathway for degradation of cotton blue

Figure 3 shows pathway for the degradation of cotton blue as model dye. HPLC, FTIR and TLC analysis confirms biodegradation of cotton blue. GC-MS and FTIR spectroscopy analysis showed triphenylmethane and sulfonamide as the final products of cotton blue degradation. Temperature, pH and biomass affected rate of decolorization. Presence of tyrosinase, lignin peroxidase and aminopyrine N-demethylase activities in the cell as well as increase in extracellular action of lignin peroxidase proposes role of these enzymes in the decolorization procedure [97].

[98], have reported that *A. flavus strain* F10 was able to reduce up to 98–99% (150 mg/L MG) malachite green after six days of incubation under optimum experimental conditions. Malachite green decolorization mechanisms approved by *A. flavus* include enzymatic reactions like hydroxylation, demethylation and ring cleavage. The immobilized growing cells are more stable and reusable than free cells for decolorization and dye degradation. Table 3 shows degradation of different dyes using fungi.

**Table 3.** Degradation of different dyes using fungi

Sr. No.	Name of dye	Dye degrading fungi	References
**1**	Malachite green	*Trichoderma sp.*	[132]
**2**	Industrial textile effluent	*Chaetomium globosum* IMA1 KJ472923	[133]
**3**	Reactive green dye [RGD]	*Fungal strain* VITAF-1	[134]
**4**	Crystal Violet and Cotton Blue	*Coriolopsis sp.*	[135]
**5**	Congo Red	*Phoma tropica MRCH* and *Dichotomomyces cejpii* MRCH 1–2	[136]
**6**	Crystal violet	*Trichoderma asperellum*	[137]
**7**	Reactive Red 31	*Aspergillus bombycis*	[138]
**8**	Reactive Violet 5 and Reactive Black 5 [diazoic]	*Trametes trogii*	[139]
**9**	Scarlet RR	*Peyronellaea* *prosopidis*	[140]
**10**	Different azo dyes	*Aspergillus flavus*	[120]
**11**	Poly R	*Pseudoglarobasidium acaciicola*	[141]
**12**	Direct Red 128 and Direct Blue	*Trametes versicolor*	[142]
**13**	Anthraquinone dye Brilliant Blue R and Remazol	*Pycnoporus sanguineus*	[143]
**14**	Textile dye	*Trametes pubescens*	[144]
	Brilliant Green	*Aspergillus sp.*	[145]
**15**	Acid Red 357 and Acid Orange 142	*Trametes villosa* SCS-10	[146]
**16**	BBKAV79 Textile industrial dyes	*Marasmius* sp.	[147]
**17**	Direct Blue-1	*Aspergillus terreus* GS28	[148]
**18**	Orange II	*T. versicolor*	[149]

#### 5.4.3. Algal biodegradation

Algae are photosynthetic microbes and they are universally spread in a wide range of surroundings. Studies reported that azo dye degradation by algae can be induced by azoreductase. Some algal species like *Oscillatoria* and *Chlorella* were able to transform toxic aromatic amines into simple metabolic intermediates like water and CO_2_ [99]. [100], have reported 98.20% and 94.19% removal of methylene blue using *C. pyrenoidosa* and *Spirulina maxima*, respectively.

#### 5.4.4. Yeast biodegradation

Numerous yeasts have been reported having ability to degrade dyes by metabolic activity such as enzymatic, biosorption, or a mixture of both [101]. Several yeasts species like *Candida tropicali, Debaryomyces polymorphous* [102], *Candida albicans* [103], and *Issatchenkia occidentalis* [104] were reported for decolorization and enzymatic biodegradation of different azo dyes. Table 4 shows degradation of different dyes using algae and yeast.

**Table 4.** Degradation of different dyes using algae and yeast

Sr. No.	Microorganism	Name of Dye	Name of Algae/Yeast	References
**1**	Algae	Direct Red-31	*Chlorella pyrenoidosa* strain NCIM 2738	[150]
		Methylene Blue	*C. pyrenoidosa*	[100]
		Methyl orange, Methylene blue, Methyl red	*Sargassum fluitanes*, *Sargassum natanes*	[151]
		Direct Red-31	*Pyrenoidosa* NCIM 2738	[150]
**2**	Yeast	Methylene Blue (MB] and Malachite Green [MG)	*Desmodesmus* sp.	[152]
		Methylene Blue	*Nostoc muscorum* [blue-green algae]	[153]
		Azo dyes	*Barnettozyma californica* strain SSA1518, Yarrowia sp. strain SSA1642, Sterigmatomyces halophilus strain SSA1511	[154]
		Reactive Black 5	*Sterigmatomyces halophilus* SSA1575	[155]

## 6. Dye industry waste and resource recovery strategy

Industrial waste can be used in resource recovery process, as input material for recovering value-added products. The main aim of these processes is to reduce the amount of waste generated from industries [105]. [106], have investigated that hybrid loose nanofilter bipolar membrane electro dialysis (BMED) procedure system can be used for removal of dye, and salt and can recycle water from dye effluent. Movable nanofiltration membrane (Ultra, Sepro NF 6) contains outstanding diafiltration performance for fractionation of (reactive and direct) dye/salt mixtures, high retention of dyes (> 99.93%) and permit the free passage of salt (R ≤ 2.2%). The addition of BMED can appreciate the making of acid or base and pure water from the salt-containing nanofillers.

Microbial fuel cell (MFC) technology is mostly used for wastewater treatment as well as energy generation. MFC technology is also used to remove odor and salinity from sea water [107]. [108], have reported use of hybrid BMED and tight ultrafiltration (TUF) procedure for resource recovery (i.e. dye extraction, pure water regeneration and, acid/base conversion) from extremely salted wastewater. They have reported that TUF membrane of 5000 Da MW can attain enough rejection rate [about >99.6%) of both direct dye and reactive dye, because of the dye collection. They have concluded that the reported procedures can be applied to sustainable management of textile wastewater.

[109], have found that commercial and sustainable chemicals can be used to recover cotton from dye wastewater. The technology involves three progressive methods: (i] leaching of textile dyes by using Nitric Acid as a pre-treatment of original waste, (ii) Dimethyl Sulfoxide (DMSO) used in dissolution procedure and (iii) bleaching procedure used diluted hydrochloric acid and sodium hypochlorite for final recovered cotton.

[118], have studied bioenergy generation and decolorization of dye by using microbial fuel cell [MFC) from dye wastewater which has shown 940.61 ± 5 mW/m^2^ power density with 790 ± 5 mV voltage output generation and 83% decolorization. [110], have reported that the methylene blue (91.9%] and CR(VI) [90.3%) recovered by using combined technical procedure with multifunctional adsorbents. [111], have reported that 66.7 ± 4.7% phosphate and 66 ± 5.3% nitrogen recovery by using multi-ion exchange membrane (IEM] stack microbial electrolysis desalination cell [MEDC). [112], have found that the maximum volumetric power density (123.2 ± 27.5 mW/m^3^] achieved by using a single-chamber microbial fuel cell (SCMFC). Other technologies like hydrothermal gasification, microbial fuel cell, constructed wetland and microbial fuel cell, etc. have been reported for degradation of dyes [Acid Orange 7, Congo Red, etc) with recovery of value-added products like bio-energy, bio-fuel etc. [113], have reported electricity generated by phytoremediation technology using microalgal strains *viz. Anabaena ambigua, Chlorella pyrenoidosa*, and *Scenedesmus abundans*.

## 7. Challenges and perspectives

Dye industrial wastewater can be used as a promising source for recovery of value-added products and treated wastewater can alos be used for agriculture applications. Overall, while dye(s] degradation using microbial system is quite promising, use of single culture is time consuming whereas; use of microbial consortium or mixed culture decreases degradation time and leads improved removal of pollutants. In this regard, membrane based processes offer potential performance benefits, especially when they are coupled with electrochemical advanced oxidation procedures such as photoelectron-catalysis, electro-Fenton and electro-catalysis, etc. However, membrane blockage and fouling still remain as major challenge when it comes to the use of pressure driven membrane process. Bio-electrochemical systems play an important role in the resource recovery process by producing clean energy, low sludge and microbial biomass.

Future studies should focus on developing pure culture as well mixed cultures-based bioremediation processes in which microbial strains should be improved for developing desirable characteristics employing tools and approaches of genetic engineering, proteomics and metabolic engineering. To fix membrane blocking and fouling issues, the research focus should be continued to develop new membranes with anti-fouling properties. Also, studies should be carried out to analyze and evaluate toxicity of degradation products due to microbial action and determine their application(s) as value-added products. Technical and economic feasibility of the treatment methods should be critically studied i.e. it is yet another way to perform in depth studies on dye degradation. Amount of the chemical used with particular textile process should be studied in detail. Scale-up of the processes with focus on resource recovery and sustainability would open up the way for integration of various technologies for treatment of dye industrial waste with simultaneous recovery of resources from it.

## 8. Conclusions

Dyes are generally released by textile, paper, pulp, tannery, cosmetic and leather industries. These types of dyes are mostly toxic when released in the environment. Substantial advancement has been made on the treatment and management of dye wastewater, which could be treated employing biological, physical and chemical methods among which biological or advanced eco-friendly methods seem most appropriate as they offer potential opportunities for resource recovery (in form of energy or chemicals) and are sustainable in nature. This review paper summarized state-of-art information and also provided scientific data that would be used for generating new opportunities in research and scientific innovations in this area.

## Supplementary Material

Supplemental Material

## Highlights

- Treatment technologies for dye industrial wastewater have been summarised.
- Recovery of resources from dye industry wastewater have been narrated.
- In depth studies are necessary to close knowledge gaps in field of bioremediation of pollutants released from dye industry
- Integration of technologies is necessary for speedy and higher remediation of pollutants.

## -

ta for this article can be accessed here
