# Supplementary material for: A critical review on advances in the practices and perspectives for the treatment of dye industry wastewater
Source: Bioengineered. 2020 Dec 28;12(1):70–87. doi: 10.1080/21655979.2020.1863034 (PMC8806354; doi:10.1080/21655979.2020.1863034)
Supplement: Supplemental Material [file KBIE_A_1863034_SM9685.zip › supplement/Highlights_Clean copy_R1.docx]

**Highlights**

- Treatment technologies for dye industrial wastewater have been summarised.
- Recovery of resources from dye industry wastewater have been narrated.
- In depth studies are necessary to close knowledge gaps in field of bioremediation of pollutants released from dye industry
- Integration of technologies is necessary for speedy and higher remediation of pollutants.
