# Supplementary figures and images for: A critical review on advances in the practices and perspectives for the treatment of dye industry wastewater
Source: Bioengineered. 2020 Dec 28;12(1):70–87. doi: 10.1080/21655979.2020.1863034 (PMC8806354; doi:10.1080/21655979.2020.1863034)

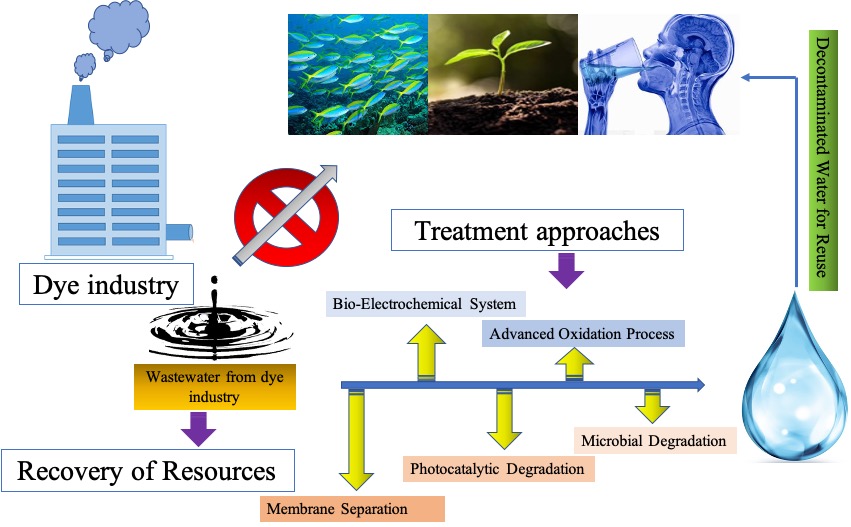

Supplement: Supplemental Material [file KBIE_A_1863034_SM9685.zip › supplement/Graphical abstract_R1.jpg]
